# International publication activity in orthopaedic surgery over a ten-year interval

**DOI:** 10.1007/s00068-024-02665-x

**Published:** 2024-09-05

**Authors:** C. Michels, K.-H. Frosch, F. T. Beil, E. S. Debus, R. T. Grundmann

**Affiliations:** 1 Department of Vascular Medicine, University Heart and Vascular Center, University Medical Center Hamburg-Eppendorf, Hamburg, Germany; 2 Department of Trauma and Orthopaedic Surgery, University Medical Center Hamburg-Eppendorf, Hamburg, Germany; 3 In den Grüben 144, Burghausen Deutschland, 84489 Germany

**Keywords:** Bibliometric analysis, Orthopaedic surgery, Impact factor, Publication activity, Benchmark

## Abstract

**Purpose:**

International English language publication activities in orthopaedic surgery comparing the years 2008/09 to 2018/19 were analyzed.

**Methods:**

20 international journals listed on PubMed were examined. The impact factor (IF) for each journal was determined using the InCites Journal Citation Report.

**Results:**

9,205 publications in 2008/09 and 15,549 in 2018/19 with 21,435 cumulative IF (CIF) in 2008/09 and 50,552 in 2018/19 were registered. Most publications consisted of narrative reviews (42.0%), followed by clinical studies (22.0%), experimental investigations (16.9%), randomized controlled trials (6.0%), and meta-analyses (5.6%). The highest increase in publications was observed for narrative reviews from 33.5% in 2008/09 to 41.1% in 2018/19. The USA had the highest number of publications (2,981 and 4,796), followed by UK (806 and 879) and Germany (606 and 922) in 2008/09 and 2018/19, resp. Per 1 Mio inhabitants, Switzerland (13.6 and 28.4), Sweden (10.9 and 18.1), the Netherlands (9.6 and 15.4), and Denmark (9.0 and 21.8) were the most productive countries in 2008/09 and 2018/19, resp.

**Conclusions:**

International publishing activity in orthopaedic surgery has increased substantially over the last 10 years. The quality of the published articles has not increased in the same way, as evidenced by the disproportionate rise in narrative reviews. Over the entire period, the US were the leader with respect to number of publications and CIF. In terms of population, however, smaller countries such as Switzerland and Sweden were much more active.

## Introduction

This study addresses international English language publication activity in orthopaedics/trauma surgery. The aim was to examine how the publication activities of countries have changed over a ten-year period (comparing the years 2008/09 to 2018/19) and which countries were leading in terms of the number of publications and impact factors (IF). This analysis not only considered the most frequently used journals but also the types of studies.

International publication activity in orthopaedics/trauma surgery was formerly analyzed over a longer period by Lee et al. [1]. They observed an increase in the number of orthopedic/trauma surgical articles published worldwide, from 2,889 in the year 2000 to 6,909 in the year 2009, with an average annual increase of 384.6 articles (10.2%). The top three countries were the USA, Japan, and the UK. The generated impact factors (IF) were not evaluated. Hohmann et al. [2] compared the publication activity in orthopaedics/trauma surgery of individual US states in 15 journals over five consecutive years (2010–2014) and correlated the activities with both the population size and the average income in those states. They found that the top four cities of New York, Philadelphia, Boston, and Chicago were responsible for 28% of the academic output over a five-year study period. The generated IF was not investigated. Furthermore, publication types (meta-analyses, randomized controlled trials, clinical studies, narrative reviews, case reports, and experimental studies) were not analyzed.

A bibliometric analysis of international publication activities over a longer period considering not only the number of publications but also their IFs and the type of publications has been presented so far only by Hinrichs et al. [3] for visceral surgery. These authors compared international publication activity in 17 English language journals over a ten-year period (years 2006/07 and 2016/17) and observed a rise in the quantity of publications by 34.8, from 2,247 in the first period and 3,029 in the last period. The generated IFs increased even more, from 9,307.6 in 2006/07 to 14,260.2 in 2016/17, a rise of 53.2%. The top five countries in 2006/07 were the USA, Japan, the UK, Germany, and Italy, and in the second period USA, Japan, the Netherlands, the UK, and France.

Our bibliometric analysis follows a similar approach for orthopaedic/trauma surgery publications. The question was: How did publication activities change with respect to the publishing countries, the most frequent journals, and publication types?

## Materials and methods

This study is based on the analysis of 20 international selected journals listed in PubMed with a focus on orthopaedics/trauma surgery, which were published in two time periods (2008/09 and 2018/19).

The selection of journals was based on two criteria: first, journals that had already been considered representative in a different context for the bibliometric analysis of trauma surgery journals were included. This included all journals selected by Hohmann et al. [2], provided they published in the years considered here and were listed in the Web of Science. These were the following 13 out of 15 journals:

- Journal of Bone and Joint Surgery American Volume (JBJS American).
- Journal of Bone and Joint Surgery British Volume / The Bone and Joint Journal (JBJS Brit / BJJ).
- American Journal of Sports Medicine (AJSM).
- Knee Surgery, Sports Traumatology, Arthroscopy (KSSTA).
- Journal of Orthopaedic Research (J Orthop Res).
- Acta Orthopaedica (Acta Orthop).
- Clinical Orthopaedics and Related Research (CORR).
- The Spine Journal (Spine J).
- Spine (Spine).
- Journal of Shoulder and Elbow Surgery (JSES).
- Injury (Injury).
- International Orthopaedics (Int Orthop).
- European Spine Journal (Eur Spine J).

Two journals were not analyzed, as one did not publish during the years considered (Journal of Arthritis), and the other could not be identified in the Web of Science (abbreviated as ASC).

Since Hohmann’s analysis focused exclusively on the activities of US academic institutions, the range of journals was to be expanded to include those that were assumed to be more frequently published by Europeans. As a result, the total number of journals analyzed increased significantly compared to Hohmann’s from 15 to 20. The selection of the latter journals was based on impact factors and editorial boards, which were intended to be international rather than US-centric. These journals were:

- Osteoarthritis and Cartilage (Osteoarthritis Cartilage).
- Arthroscopy: The Journal of Arthroscopic Related Research (Arthroscopy).
- Journal of Orthopaedics and Sports Physical Therapy (JOSPT).
- The Journal of Arthroplasty (J Arthroplasty).
- Archives of Orthopaedic and Trauma Surgery (Arch Orthop Trauma Surg).
- BMC Musculoskeletal Disorders (BMC).
- European Journal of Trauma and Emergency Surgery (Eur J of Trauma Emerg Surg).

Only printed articles with an abstract available on PubMed were included. To assign a publication to a country of origin, the nationality of the authors was checked. For publications originating from a single institution, there was only one author and one country. In the case of multinational publications, the publication was attributed to all nations involved in the publication. The number of publishing countries was then higher than the number of analyzed publications. The analyzed publications were categorized into meta-analyses, clinical studies, randomized controlled trials (RCTs), case reports, experimental investigations, narrative reviews, and other types.

The cumulative impact factors for journals and countries in each time period were calculated. The IF for each journal was determined using the InCites Journal Citation Report by Clarivate Analytics.

For this investigation, the authors did not conduct any studies on humans or animals.

## Results

### Number of publications in individual journals

A total of 9,205 publications were published in 2008 and 2009. The top three journals during this period were “Spine” with 1,218 publications (13.2% of all publications during this time frame), “CORR” with 776 publications (8.4%), and “JBJS American” with 560 publications (7.1%).

There were significant shifts in 2018/2019 in comparison to 2008/09, as shown in Table 1. In the last period, a total of 15,549 publications were published, representing a 68.9% increase compared to 2008/2009. The top three journals in 2018/2019 were “J Arthroplasty” with 1,452 publications (9.3%), “KSSTA” with 1,390 publications (9.0%), and “BMC” with 1,289 publications (8.3%).

**Table 1:** Number of publications and cumulative impact factors (CIF) of the 20 journals for the analyzed years. Sequence according to the total number of publications

Journal	2008/2009 *n* (%)	2018/2019 *n* (%)	2008/2009 CIF (%)	2018/2019 CIF (%)
Spine	1,218 (13.2)	1,061 (6.8)	3,299 (15.4)	2,953 (5.8)
J Arthroplasty	505 (5.5)	1,452 (9.3)	845 (3.9)	5,244 (10.4)
KSSTA	411 (4.5)	1,390 (8.9)	692 (3.2)	4,389 (8.7)
BMC	335 (3.6)	1,289 (8.3)	648 (3.0)	2,479 (4.9)
Eur Spine J	491 (5.3)	982 (6.3)	1,059 (4.9)	2,447 (4.8)
Injury	509 (5.5)	959 (6.2)	1,105 (5.2)	2,028 (4.0)
AJSM	448 (4.9)	988 (6.4)	1,622 (7.6)	5,859 (11.6)
JBJS American	650 (7.1)	666 (4.3)	2,192 (10.2)	3,094 (6.1)
J Orthop Res	470 (5.1)	837 (5.4)	1,430 (6.7)	2,431 (4.8)
Int Orthop	403 (4.4)	787 (5.0)	662 (3.1)	2,055 (4.1)
CORR	776 (8.4)	400 (2.6)	1,537 (7.2)	1,302 (2.6)
Arthroscopy	398 (4.3)	761 (4.9)	1,016 (4.7)	3,335 (6.6)
JSES	322 (3.5)	833 (5.4)	604 (2.8)	2,368 (4.7)
JBJS Brit/ BJJ	567 (6.2)	530 (3.4)	1,374 (6.4)	2,281 (4.5)
Osteoarthritis Cartilage	416 (4.5)	538 (3.5)	1,657 (7.7)	2,603 (5.1)
Spine J	303 (3.3)	623 (4.0)	488 (2.3)	1,990 (3.9)
Arch Orthop Trauma Surg	231 (2.5)	530 (3.4)	399 (1.9)	1,059 (2.1)
Eur J Trauma Emerg Surg	215 (2.3)	314 (2.0)	23 (0.1)	627 (1.2)
Acta Orthop	231 (2.5)	282 (1.8)	425 (2.0)	871 (1.7)
JOSPT	164 (1.8)	327 (2.1)	360 (1.7)	1,137 (2.3)
**Sum**	**9**,**205 (100)**	**15**,**549 (100)**	**21**,**435 (100)**	**50**,**552 (100)**

#### Number of cumulative impact factors (CIF) by individual journals

In the years 2008 and 2009, a total of 21,435 IF were cumulated. The top three journals during the 2008 and 2009 period were “Spine” with 3,298 CIF (15.4%), “JBJS American” with 2,191 CIF (10.2%), and “Osteoarth Cartilage ” with 1,657 CIF (7.7%).

In 2018/19, a total of 50,552 IF were cumulated (Table 1), representing a 135.8% increase compared to 2008/2009. The top three journals in the last period were “AJSM” with 5,859 CIF (11.6% of all CIF), “J Arthroplasty” with 5,244 CIF (10.4% of all CIF), and “KSSTA” with 4,389 CIF (8.7% of all CIF).

#### Publication activities of the 14 most-publishing countries

The top three countries during the first analyzed time period were the USA with 2,981 publications (32.4%), the UK with 806 publications (8.8%), and Germany with 606 publications (6.6%).

There were shifts in the ranking of the publishing countries in 2018/2019, as shown in Table 2. The USA still led with 4,796 publications (30.8%), China was now in second place with 1,126 publications (7.2%), and Germany maintained its third position with 922 publications (5.9%). United Kingdom held now the fourth position.

**Table 2:** Number of publications and cumulative impact factors (CIF) of the 14 most publishing countries for the analyzed years. Sequence according to the total number of publications

Country	2008/2009 *n* (%)	2018/2019 *n* (%)	2008/09 CIF (%)	2018/19 CIF (%)
USA	2,981 (32.4)	4,796 (30.8)	7,633 (35.6)	17,324 (34.3)
UK	806 (8.8)	879 (5.7)	1,776 (8.3)	2,864 (5.7)
Germany	606 (6.6)	922 (5.9)	1,202 (5.6)	2,629 (5.2)
China	347 (3.8)	1,126 (7.2)	892 (4.2)	3,209 (6.4)
Japan	579 (6.3)	842 (5.4)	1,352 (6.3)	2,674 (5.3)
South Korea	407 (4.4)	643 (4.1)	930 (4.3)	2,119 (4.2)
Canada	386 (4.2)	640 (4.1)	982 (4.6)	2,142 (4.2)
Australia	267 (2.9)	593 (3.8)	607 (2.8)	1,940 (3.8)
Netherlands	318 (3.5)	531 (3.4)	636 (3.0)	1,654 (3.3)
Switzerland	210 (2.3)	485 (3.1)	461 (2.2)	1,467 (2.9)
Italy	219 (2.4)	435 (2.8)	475 (2.2)	1,288 (2.5)
France	180 (2.0)	448 (2.9)	421 (2.0)	1,382 (2.7)
Sweden	202 (2.2)	371 (2.4)	446 (2.1)	1,161 (2.3)
Denmark	99 (1.1)	253 (1.6)	229 (1.1)	812 (1.6)
All other countries	1,598 (17.4)	2,585 (16.6)	3,393 (15,8)	7,887 (15,6)
**Sum**	**9**,**205 (100)**	**15**,**549 (100)**	**21**,**435 (100)**	**50**,**552 (100)**

“All other countries” indicates 56 countries

### Number of cumulative impact factors (CIF) by countries

In the years 2008 and 2009, the top three countries were the USA with 7,633 CIF (35.6%), the UK with 1,776 CIF (8.3%), and Japan with 1,352 CIF (6.3%).

In 2018/19, the USA held still the first position with 17,324 CIF (34.3%), followed now by China with 3,209 CIF (6.4%), and the UK with 2,864 CIF (5.7%) (Table 2).

### Types of publications

Narrative reviews were the dominating publication type in both analyzed time periods, increasing from 33.5% to 47.1% of all publications (Table 3). Meta-analyses experienced a notable increase, accounting for only 2.3% in 2008/2009, but for 7.5% of all publications ten years later.

**Table 3:** Types of publications in 2008/09 and 2018/19. Sequence according to the total number of publications

Type of publications	2008/2009 *n* (%)	2018/2019 *n* (%)
Narrative reviews	3,081 (33.5)	7,320 (47.1)
Clinical studies	2,269 (24.6)	3,187 (20.5)
Experimental studies	1,975 (21.5)	2,206 (14.2)
RCT	575 (6.2)	908 (5.8)
Meta-analysis	209 (2.3)	1,171 (7.5)
Case reports	850 (9.2)	417 (2.7)
Other	246 (2.7)	340 (2.2)
**Sum**	**9**,**205 (100)**	**15**,**549 (100)**

RCT: randomized controlled trails

#### Types of publication per country

Table 4 presents the types of publication for the five most publishing countries in both analysed periods. In general, narrative reviews were the most published types, with an obvious increase in 2018/19 compared to 2008/09. However, an increase in meta-analyses is noticeable, where experimental studies, case reports and “others” are decreasing.

**Table 4:** Types of publication in the five most publishing countries. The percentages refer to the total number of publications of each country

		Narrative Reviews *n* (%)	Clinical Studies *n* (%)	Experimental Studies *n* (%)	RCTs *n* (%)	Meta-analyses *n* (%)	Case Reports and “others” *n* (%)
2008/09	USA	1,115 (37.4)	569 (19.1)	788 (26.4)	145 (4.9)	66 (2.2)	298 (10.0)
	UK	287 (35.6)	225 (27.9)	108 (13.4)	45 (5.6)	30 (3.7)	111 (13.8)
	GER	155 (25.6)	172 (28.4)	157 (25.9)	42 (6.9)	6 (1.0)	74 (12.2)
	China	92 (26.5)	88 (25.4)	103 (29.7)	18 (5.2)	10 (2.9)	36 (10.4)
	Japan	157 (27.1)	124 (21.4)	174 (30.1)	17 (2.9)	2 (0.4)	105 (18.1)
2018/19	USA	2,429 (50.7)	831 (17.3)	751 (15.6)	195 (4.1)	342 (7.1)	248 (5.2)
	UK	399 (45.4)	195 (22.2)	104 (11.8)	50 (5.7)	90 (10.2)	41 (4.7)
	GER	409 (44.4)	214 (23.2)	178 (19.3)	51 (5.5)	37 (4.0)	33 (3.6)
	China	524 (46.5)	151 (13.4)	217 (19.3)	60 (5.3)	97 (8.6)	77 (6.8)
	Japan	429 (51.0)	164 (19.5)	163 (19.4)	29 (3.4)	11 (1.3)	46 (5.5)

RCT: Randomized control trial; GER: Germany

#### Ranking by number of RCTs and meta-analyses

RCTs, meta-analyses and systematic reviews represent the study designs with the highest level of evidence [7]. In 2008/09, the USA, UK and the Netherlands occupied the top three positions. Ten years later, the USA, China and Canada were the leading countries (Table 5).

**Table 5:** Top 11 countries ranked by total number of meta-analyses and RCTs in 2008/2009 compared to 2018/2019

2008/2009		2018/2019	
Country	*n* (%)	Country	*n* (%)
USA	211 (26.9)	USA	537 (25.8)
UK	75 (9.6)	China	157 (7.6)
Netherlands	59 (7.5)	Canada	141 (6.8)
Canada	56 (7.1)	UK	140 (6.7)
Germany	48 (6.1)	Netherlands	121 (5.8)
Sweden	39 (5.0)	Australia	121 (5.8)
Australia	37 (4.7)	Germany	87 (4.2)
China	28 (3.6)	South Korea	74 (3.6)
Denmark	22 (2.8)	Italy	64 (3.1)
Italy	20 (2.6)	Switzerland	58 (2.8)
Japan	19 (2.4)	Sweden	58 (2.8)
All other countries	170 (21.7)	All other countries	521 (25.1)
**Total**	**784 (100)**	**Total**	**2**,**079 (100)**

“All other countries” indicates 59 countries

### Population-based publication activities and cumulative impact factors (CIF)

The publication activity per one million inhabitants of the 14 most-publishing countries is presented in Table 6. Switzerland ranked first in both study periods with 13.6 publications per one million inhabitants in 2008/2009 and 28.4 publications per one million inhabitants in 2018/2019. In the 2008/2009 period, Sweden, the Netherlands, and Denmark followed, and these countries also remained among the top performers in 2018/2019.

**Table 6:** Population-based publishing activities and cumulative impact factors (CIF) of the 14 most publishing countries for each time period

2008/2009			2018/2019		
Country	Publications/ 10⁶ Inhabitants (*n*)	CIF/ 10⁶ Inhabitants (*n*)	Country	Publications/ 10⁶ Inhabitants (*n*)	CIF/ 10⁶ Inhabitants (*n*)
Switzerland	13.6	29.9	Switzerland	28.4	85.9
Sweden	10.9	20.8	Denmark	21.8	70.0
Netherlands	9.6	24.1	Sweden	18.1	56.8
Denmark	9.0	21.4	Netherlands	15.4	47.8
UK	6.5	14.8	Australia	11.8	38.6
Australia	6.2	14.3	Canada	8.6	28.7
Canada	5.8	14.7	USA	7.3	26.4
USA	4.9	12.5	UK	6.6	21.5
South Korea	4.1	7.3	South Korea	6.2	20.5
Germany	3.7	9.5	Germany	5.6	15.8
Japan	2.3	5.3	Italy	3.6	10.7
Italy	1.9	4.0	France	3.3	10.6
France	1.4	3.3	Japan	3.3	10.3
China	0.1	0.3	China	0.4	1.1

The cumulative impact factors (CIF) of the analyzed countries per one million inhabitants are also shown in Table 6. Switzerland, Sweden, the Netherlands, and Denmark led in this category as well. This ranking was consistent for both the 2008/2009 and 2018/2019 periods.

## Discussion

This study found an increase of publication activity in orthopaedic surgery of 68.9% over a ten-year period. Sun et al. [4] also examined the publication activities of orthopedic journals, focusing on the years 2017–2020. They described an increase of publications by 37.4% over the analyzed time, which is significantly lower, probably because of the shorter analyzed period.

Hohmann et al. [2] investigated publication frequency in orthopaedics/trauma surgery in the USA across 15 selected journals, analyzing 8,100 articles over a four-year period (2010–2014). The three most publishing journals were “JBJS American”, “CORR”, and “Spine”. This only partially corresponds to the results presented in our study. Only “Spine” was also among the three most publishing journals with 2,279 publications in all four years.

In our study, in the years 2018/2019, “J Arthroplasty” published 1,452 articles, “KSSTA” published 1,390 articles, and “BMC Musculoskeletal Disorders” published 1,289 articles, making them the leading journals in this time period with an increase of 187.5%, 238.2%, and 284.8% respectively compared to 2008/2009. In contrast, there was a significant decline in publications for “Spine”, from 1,218 to 1,061 articles, and for “CORR”, from 776 to 400 articles, representing a decrease of 12.9% and 48.5% respectively in the same period. But overall, an increase in publications was observed (68,9%). Loder et al. [8] also examined publication frequency in individual journals based on the number of manuscripts. When comparing 2015/2016 with 2005/2006, they found an increase in publications over a decade (BONE, JOR, AJSM, Injury, JAR, JHSA, JOT) in seven out of 17 journals, five journals showed no significant changes, and five journals had fewer manuscripts (Arthroscopy, BJJ, FAI, JBJS, Spine).

Lee et al. [1] examined trends in publication frequency based on a total of 46,322 orthopedic/trauma articles published from 2000 to 2009. They observed an increase in the annual global publication of orthopedic/trauma articles from 2,889 in 2000 to 6,909 in 2009, with an average annual increase of 384.6 articles (10.2%). In this study, an increase from 9,205 to 15,549 articles over ten years was observed, which would theoretically represent an increase of 634.4 more articles per year.

Lee et al. [1] also analyzed publications by national origin. In descending order, the six most internationally publishing countries were in 2000 the USA, followed by Japan, the UK, China, Germany, and Korea. This order had shifted by 2009, with Korea now placed fourth and China sixth. The results of our study largely align with those. In 2018/2019, the same countries mentioned by Lee et al. [1] were among the top five, although in a different order, with the USA in the first place, followed by China, Germany, the UK, and Japan.

Hohmann et al. [5] analyzed 23,021 orthopaedic publications from 66 countries in 15 journals over a four-year period (2010 to 2014). The top three countries with the highest publication count were the USA, the UK, and Japan. The highest sum of impact factors was achieved by the USA, the UK, and Japan. These results correspond to this analysis, where the USA and the UK were also the most publishing countries.

Zhi et al. [9] also examined the publication activities of six individual countries based on a total of 143,138 orthopedic/trauma surgery articles published between 2005 and 2014. They only presented growth rates graphically, making it difficult to determine exact figures. As Chinese authors, they were primarily interested in precise data for China and reported that China contributed 2.9% of the total number of publications, compared to 24.9% for the USA, followed by the UK with 5.5%. The authors did not specify whether these were exclusively English-language journals and articles. Therefore, this study is not directly comparable with our analysis.

A bibliometric analysis by Mani et al. [10] focused on the orthopedic/trauma surgery publication frequency in 41 European countries that are members of EFORT. In that study, the UK, Italy, and Germany were the leading countries. In our European ranking the UK, Germany, and the Netherlands occupied the top positions, with Italy ranking fifth.

In this study, countries were also ranked by their cumulative impact factors (CIF). There were hardly any differences between the country rankings based on CIF compared to publication activity. The top four countries by CIF were the USA, the UK, Japan, and China.

Only Hohmann et al. [5] conducted a ranking of countries based on impact factors in the field of orthopaedic surgery. In their study, the top five countries accounted for 60.4% of all publications (*n* = 23,021) and 61.4% of all CIF (*n* = 66,496.7), which is nearly consistent with our analysis. In this study, the top five countries were responsible for 56.1% of all publications (*n* = 24,754) and 57.6% of all CIF (*n* = 71,987).

Publications in orthopaedics/trauma surgery were here differentiated into meta-analyses, clinical studies, randomized controlled trials, case reports, narrative reviews, and experimental studies (Table 3). We did not find a similar study on this topic. A significant decrease in published case reports by 50.9% was observed when comparing the years 2008/2009 to 2018/2019, while simultaneously, the impact factor of all analyzed journals increased. As the overall publication count increased, case reports accounted for only 2.7% of all publications in the second period, compared to 9.2% in the first period. Erivan et al. [6] analyzed only the impact of case reports on journal’s impact factor in orthopaedics/trauma surgery for the years 2015–2017. They described a decrease in the impact factor of scientific journals due to the publication of case reports. They calculated that 69 out of 79 analyzed journals would have had a higher impact factor, if they had completely avoided publishing case reports.

The increase in English-language publications observed by Hohmann et al. [5] for the years 2010 to 2014 was also confirmed in our analysis, comparing publications in 2008/09 and 2018/19 within a larger number of journals. An increase in the number of publications does not by itself indicate an improvement in quality, as does not an increase in impact factors (IFs) alone. Therefore, in this study for the first time the type of publications (meta-analyses, RCTs, clinical studies, case reports, etc.) has been examined. This is a crucial prerequisite for quality assessment, as the increase in impact factors (IFs) alone is insufficient, especially since the IF of all journals has risen equally during the observed period. While the number of RCTs and meta-analyses increased during the observation period, the greatest percentage increase was seen in narrative reviews, which accounted for almost half (47.1%) of all publications in the second observation period, but this does not demonstrate an improvement in publication quality. If one aims to present the publication quality of a country in an international comparison, rather the number of meta-analyses and RCTs should specifically be considered. For example, the USA published 30% of the total publications but only 25% of the meta-analyses and RCTs. In contrast, Canada, the UK, and the Netherlands accounted for 4.1%, 5.7%, and 3.4% respectively of the total publications, but 6.8%, 6.7%, and 5.8% respectively of the meta-analyses and RCTs. This indicates that the share of the latter countries in qualitative publications is higher than their percentage of the total number of publications.

Furthermore, a population-based ranking should be performed for every bibliometric comparative analysis in order to assess the performance of academic institutions and countries. In our analysis, the USA, the UK, Germany, and China ranked highest in terms of publication count. However, when considering population-based metrics, entirely different countries were leading, including Switzerland, Sweden, Denmark, and the Netherlands for both the 2008/2009 and 2018/2019 periods.

This trend aligns with an analysis by Lee et al. [1]. In that study, the USA, Japan, the UK, and Germany were the leading countries in terms of publication count in 2008 and 2009, while population based rankings included Switzerland, the UK, and Israel among the top performers.

Hohmann et al. [5] also examined each country’s publication performance in orthopaedics based on population in the period from 2010 to 2014. In terms of publication count, they identified the USA, the UK, Japan, South Korea, and Germany as the most publishing countries. However, in population-based rankings, Switzerland, Norway, Denmark, Sweden, and the Netherlands led. This corresponds with our study, where Switzerland and Scandinavian countries also ranked highest.

It seems quite clear that countries like Germany, Italy, and France do not have the same incentives to engage in scientific work compared to Switzerland, the Scandinavian countries, and the Netherlands. The reasons for this should be further analysed.

### Limitations

One limitation of this study was the decision to select and analyze a specific number of journals (*n* = 20). For comparative purposes, 13 out of the 15 journals were chosen, which were also considered by Hohmann et al. [2]. The additional journals analyzed were selected based on their impact factor, ensuring that at least the most widely used English language journals in orthopaedics/trauma surgery were included. Therefore, it can be assumed that the 20 selected journals were representative for English-language orthopaedics/trauma surgery.

The fact that only English language publications were included in this study limits the ranking of non-English speaking countries.

Other limitations arise from the fact that only journals listed in PubMed were analyzed, meaning that non-PubMed-listed journals had no influence on the study results.

## Conclusions

Our investigation highlights the significantly increasing English-language international publication activity from 2008/09 to 2018/19. This applies to both the number of publications and the impact factors of individual journals. The quality of the published articles has not increased in the same way, as evidenced by the disproportionate rise in narrative reviews from 33.5% to 47.1% of the total number of publications. Bibliometric analyses allow for the assessment of the scientific output of individual countries over time in an international comparison. However, population-based activities must also be considered: The USA were the most productive country in total number of publications, however, smaller countries were more active in relation to their population size.

## Data Availability

No datasets were generated or analysed during the current study.
